# *P* values in display items are ubiquitous and almost invariably significant: A survey of top science journals

**DOI:** 10.1371/journal.pone.0197440

**Published:** 2018-05-15

**Authors:** Ioana Alina Cristea, John P. A. Ioannidis

**Affiliations:** 1 Meta-Research Innovation Center at Stanford (METRICS), Stanford University, Stanford, California, United States of America; 2 Department of Clinical Psychology and Psychotherapy, Babes-Bolyai University, Cluj-Napoca Romania; 3 Departments of Medicine, Stanford University, Stanford, California, United States of America; 4 Department of Health Research and Policy, Stanford University, Stanford, California, United States of America; 5 Department of Biomedical Data Science, Stanford University, Stanford, California, United States of America; 6 Department of Statistics, Stanford University, Stanford, California, United States of America; CPERI, GREECE

## Abstract

*P* values represent a widely used, but pervasively misunderstood and fiercely contested method of scientific inference. Display items, such as figures and tables, often containing the main results, are an important source of *P* values. We conducted a survey comparing the overall use of *P* values and the occurrence of significant *P* values in display items of a sample of articles in the three top multidisciplinary journals (Nature, Science, PNAS) in 2017 and, respectively, in 1997. We also examined the reporting of multiplicity corrections and its potential influence on the proportion of statistically significant *P* values. Our findings demonstrated substantial and growing reliance on *P* values in display items, with increases of 2.5 to 14.5 times in 2017 compared to 1997. The overwhelming majority of *P* values (94%, 95% confidence interval [CI] 92% to 96%) were statistically significant. Methods to adjust for multiplicity were almost non-existent in 1997, but reported in many articles relying on *P* values in 2017 (Nature 68%, Science 48%, PNAS 38%). In their absence, almost all reported *P* values were statistically significant (98%, 95% CI 96% to 99%). Conversely, when any multiplicity corrections were described, 88% (95% CI 82% to 93%) of reported *P* values were statistically significant. Use of Bayesian methods was scant (2.5%) and rarely (0.7%) articles relied exclusively on Bayesian statistics. Overall, wider appreciation of the need for multiplicity corrections is a welcome evolution, but the rapid growth of reliance on *P* values and implausibly high rates of reported statistical significance are worrisome.

## Introduction

The long-standing controversy over how best to make inferences from empirical data is intricately related to the notion of “statistical significance”. The most widespread markers of statistical significance are constituted by *P* values derived from null hypothesis significance testing. *P* values indicate “the probability that a chosen test statistic would have been at least as large as its observed values if every model assumption were true, including the test hypothesis.” [1] (p.339). In particular, using the *P* = .05 cut-off for separating statistically significant from non-significant findings [2] has been widely adopted and embraced as a tool for deciding whether a research finding is “true, valid and worth acting on” [3]. One of the most widespread misunderstandings of *P* values is the notion they “measure the probability that the studied hypothesis is true” [4] (p.131).

Warnings of pervasive misunderstanding of what *P* values show and how they can be used [1, 5–9] have given way to fierce debate over their having any usefulness at all [2, 10–12], prompting some scientists to coin the term “*P* value wars” [13]. Nonetheless, *P* values remain ubiquitous in biomedical and social science research. A recent large-scale evaluation [14] showed that the proportion of articles in PubMed that use *P* values either in the abstract or in the full-text is increasing over time. Moreover, among the papers that used *P* values, 96% reported at least one such value of .05 or lower [14]. This was equally true for the abstracts of papers and for the full texts [14]. There was a slight decrease in this proportion overtime between 1990 and 2014 from 98% to 95%. In-depth full-text analysis showed over 55% of randomly sampled articles reported at least one *P* value [14]. Another recent evaluation showed a concomitant decrease in effect size (ES) values and increase in the proportion of statistically significant ESs over time [15].

One plausible reason for the increase in reported P values and particularly statistically significant ones might also be attributed to the unprecedented computational facility afforded by widely used statistical software. Hence, researchers have the possibility of running a myriad of tests and obtaining many low P values by chance alone. Certain fields, such as genetics, neuroimaging and omics are particularly exposed to this problem. There is no one size fits all recommendation to tackle this problem and different fields and different types of research might take up various approaches. One solution proposed was lowering the *P* value threshold, already adopted by some fields [16, 17] and debated in others [18–20]. Another partial solution was employing various methods to correct for multiple testing, a topic on which there is a vast and nuanced scholarship related to the scope, benefits and caveats of these methods [21–28].

One prominent alternative paradigm to the use *P* values involves Bayesian methods. Calls for including Bayesian inference methods in addition or in lieu of traditional significance testing have become more frequent [3, 6, 29, 30], and potential benefits for their application to various strains of basic [31–33] and applied [34–36] research have been demonstrated. For the implementation of Bayesian analyses, researchers can also count on a growing number of methodological resources [37] and software packages, both dedicated (e.g., JASP, Stan, WinBUGS), as well as modules integrated in general purpose statistical software (SAS, STATA).

Most research [38–42] on *P* values has focused on their distribution across subsets of the literature, as a potential indicator of publication bias either by suppressing non-significant results completely (“the file drawer”) or by selectively reporting only the best looking significant ones, usually after having tried various types of analysis and model specifications (e.g., “*P* hacking” [43]). A related, under-studied aspect refers to their proportion over time, both as the total collection of *P* values of different magnitudes, as well as those formally defined as statistically significant, most often through the *P*< .05 decision rule. Though estimates of this have been obtained from abstracts [14, 15, 44] and random samples of full-text [14, 15], display item such as figures and tables have been scarcely studied. Text mining approaches used in previous studies typically are unable to scan *P* values that are embedded in tables or figures [14, 15, 41, 42]. Nevertheless, these display items often contain the main results of the paper, particularly in highly influential journals looking to chiefly publish cutting-edge articles. In these top journals, strict word count limitations imply that most articles are published in brief formats (e.g., letter for Nature) and most results are compressed in the figures. These are generally complex and composed by an array of different graphical elements, with only a small fraction of the results displayed being highlighted in the text. Like abstracts, figures are often used to synthesize the key findings of the paper in the public reception of the paper in the media or the community. They are also likely to contain more complete results than those that are selectively highlighted in the text and those that are even more heavily selected for presentation in the abstract [45].

Here, we conducted a survey comparing the overall use of *P* values and the occurrence of statistically significant *P* values in display items of a sample of articles in the three top multidisciplinary journals (Nature, Science, PNAS) and compared the current situation (for articles published in 2017) with that of 20 years ago. We explored whether the use of *P* values in display items was also accompanied by an augmented use of methods to correct for multiple testing. We also examined whether alternative approaches to data analyses, such as Bayesian methods, were used and were becoming more frequent in these top journals.

## Method

### Selection of journals and issues for inclusion

Nature, Science and PNAS were chosen as the top multi-disciplinary journals, covering all fields of science. These are generally acknowledged as extremely prestigious outlets for research, as publishing in any of them weighs greatly for a researcher’s career (e.g., grant applications, promotion, tenure).

We randomly chose January as our month of interest and selected issues published in the months of January 2017, and respectively in January 1997. Four published issues were considered for Nature and Science. Given that regular issues of PNAS contain a considerably larger number of articles, only one issue per year was included for PNAS, so as to enhance comparability. The order in which journals were screened was Nature, Science, PNAS and for each we first screened 2017, starting from the 1^st^ published issue of the year.

### Selection of papers and display items

For each issue, all published research articles were considered for inclusion. These usually included full length articles and briefer reports, communications and research letters. Policy articles, news, editorials, reader correspondence and other items outside of regular peer-review were excluded, as were reviews, opinion and other type of data-free pieces. Research articles without any display items were also excluded.

### Data extraction

One researcher (IC) manually screened each research article to establish if it contained any display items. For each display item (table or figure), *P* value information was extracted both from the main corpus, as well as from the legend. The researcher manually counted the number of total *P* values and the number of significant *P* values. *P* values had to specifically labelled as such with the letter “P” or “p” and described, either by giving exact values, or by marking (e.g., with a symbol), or by indicating they represented values smaller or larger than a specified threshold. Figures reporting inferential parameters of another nature (e.g. False Discovery Rate corrected q values or E-values) labelled as such were not counted. General phrases in the legend such as “no changes” or “no significant changes” were not counted since it was unclear whether they were referring to non-significant *P* values and, if so, to how many of these. Similarly, phrases like “significant changes” in figure legends, not accompanied by specific *P* values, were also not counted.

For each article, we extracted the total number of display items, the total number of display items containing *P* values, the total number of countable *P* values, and the total numbers of statistically significant ones, following the authors’ own definition of statistical significance. We also noted whether any type of correction for multiplicity was mentioned in the display item. However, sometimes authors might have performed these corrections but only mentioned them in the Methods section. Therefore, for the articles in which at least one display item contained *P* values, we perused the Methods section, both in the main text and supplementary material and extracted information about the use of any statistical method to correct for multiple tests or comparisons. In case nothing was found, we also perused the supplementary figures for any similar hints on multiplicity correction.

We expected the articles to cover a wide range of scientific fields and consequently deemed it impossible to be able to fully and accurately understand the full complexity of the analyses presented. Consequently, we opted for an approach of tallying *P* values by taking everything presented in the display item at face value and staying clear from recording what exactly was being compared. Any graphic element such as a bar plot that was not marked with a *P* value or symbol representing a *P* value was not counted. We first counted *P* values from the body of the display item and then checked the legend to whether there were any additional *P* values, except those mentioned in the body. In this way, we assured that, while taking the figure legends into account, we would not inadvertently count any *P* values twice. In the cases of papers using more complex notations, like different letters to indicate statistically significant differences, we opted for the simplest, most direct method of tallying and avoided having to infer how many comparisons had been or could have been performed. More specifically, in bar graphs marked with letters whereby the figure legend specified different combinations of letters indicated various types of statistical significance we used the following sequence of rules: (a) if there were one or more panels where pairs of bars were marked with a line on top or an underscore, alongside with letters, we counted these pairs as *P* value units; if the letters on 2 such combined bars were different, this was considered as one significant *P* value; (b) if there were more panels containing bars not marked otherwise except for a letter on each bar, we considered each panel as a *P* value unit; if any panel contained at least 2 bars with different letters, that was counted as one significant *P* value; and finally (c) in the presence of just one panel, we considered all possible permutations between 2 bars as *P* values; any permutation where the letters of 2 bars were different was considered as a significant *P* value. In deciding between rules a, b or c, we were also mindful of what the authors specified in the figure legend (i.e., if they gave a clear indication of how comparisons between bars were considered). In some types of graphic elements, such as scatter plots, Manhattan plots, volcano plots, brain activation maps, heat maps and others, the number of *P* values was impossible to count manually. In those cases, we recorded how many such graphic elements were in the paper; moreover, if the display item contained other countable *P* values in other panels, those were counted. If there were no such sections, the display item was marked as having *P* values, but entirely impossible to count. Bar graphs of log-transformed *P* values for a set of variables or similar were only counted if we could unequivocally place the statistical significance line (either it was marked by the authors or we used a ruler) so as to determine for which variables it was located under the .05 threshold. Papers mentioning Bayesian methods in the display items were counted separately, regardless of whether or not they also presented any *P* values; if they did contain *P* values as well, these were tabulated as above.

In the case of uncertainty about extracting *P* value counts or about whether a method described was a multiplicity adjustment, the other researcher (JPAI) was consulted, and independently extracted the information. Disagreements were resolved by discussion.

### Data synthesis

Each journal accompanied by the year was considered a unit (e.g., Nature 2017). Our main outcomes of interest were the total number of *P* values as well as the ratio of significant to total *P* values, per Journal-Year unit.

For each unit, we computed the total number of research articles screened, the total number of articles with display items, the total number of display items, the total number of number of display items with completely uncountable and, respectively, countable *P* values, the total number of *P* values and, out of these, the total number of significant *P* values. For each unit, we also noted the total number of articles presenting results with Bayesian methods. In a second step, for all articles containing at least one display item with countable *P* values, we extracted and tabulated the information about any multiplicity corrections performed.

Results were synthesized descriptively, by reporting total counts, medians, IQRs, percentages and 95% confidence intervals, as well as by use of meta-analysis of proportions. In this meta-analysis, we aggregated the proportion of significant to total *P* values for each display item. We expected that some articles and particularly some display items would include a large number of *P* values and so in an overall estimate, they would necessarily have to be weighted more. For the individual display items, the Clopper-Pearson exact method [46], which inverts the equal-tailed test based on the binomial distribution, was used to determine confidence limits for the proportions of significant to total P values. As we expected many ratios of significant to total *P* values to be close to 1, we employed the Freeman-Tukey double arcsine transformation to stabilize the variance [47] of the individual ratios. The pooled estimate for the overall proportion of significant to total *P* values was then computed using the transformed values and their variances with the inverse-variance DerSimonian and Laird method [48]. The confidence intervals for the pooled estimate are computed using the Wald method. All analyses were implemented in STATA [49], using the package *Metaprop_one* for meta-analysis [50].

We expected a major methodological shift from 1997 to 2017, in particular with regards to the use of multiplicity corrections, which have become more standard over time, as the problem of multiple testing became widely recognized and various methods were proposed and more widely adopted. Corrections for violations of normality or homogeneity of variance, and the standard *post-hoc* corrected tests used as part of an ANOVA were not considered as adequate multiplicity adjustments, as these either address other aspects in the data, or are an integral part of the statistical procedure for a testing a single hypothesis, which could nonetheless be repeated multiple times.

We conducted subgroup analysis comparing the proportion of statistically significant *P* values (1) across all Journal-Year units; (2) comparing the three journals stratified by year, in 2017, and respectively 1997; (3) between articles employing versus non-employing adequate multiplicity corrections, across all units; (4) comparing the three journals across articles employing and respectively not-employing proper multiplicity corrections in 2017 (we expected to have a reduced number of articles using such corrections in 1997). We used Bonferroni multiplicity correction to consider subgroup differences to be statistically significant at p<0.008 (0.05/6). We also conducted robustness analyses excluding the display items in which we were unsure about counting the total or statistically significant *P* values and therefore our calculations could have been inaccurate.

## Results

### Total articles and display items with *P* values (Table 1)

Four published issues, covering the whole month of January were considered for Nature and Science in 2017. Since January 1997 had 5 weeks, four out of the five published issues were considered. The excluded issues were the last monthly issue of Nature (issue 6616) and a special issue (issue 5298 Bioinformatics) for Science. We screened a total of 409 articles and 390 were eligible. The total eligible articles for Nature, Science and PNAS were 65, 62 and 51 in 2017 and 78, 61 and 54 in 1997, respectively. Overall, the articles contained a total of 1504 display items, distributed fairly symmetrically across journals and years, ranging from 204 (Nature 2017) to 284 (PNAS 2017). 110 articles (27% of all articles) included 287 display items containing *P* values (19% of all displays). In 2017, a similar number of such articles (around 20) was present in each journal and 39% of all articles contained displays with *P* values. For 1997, the estimation was similar for Nature, but approximately 3 times smaller for Science and PNAS (9 and respectively 8 articles) and overall only 21% of all articles contained displays with P values. For 8 out of the 287 display items (7 of them published in 2017), no *P* values were manually countable due to the type of graph (e.g., volcano plot). The total number of display items with some countable *P* values increased two-fold from 1997 to 2017 for Nature (36 versus 74), three-fold for Science (18 versus 60) and seven-fold for PNAS (12 versus 87).

**Table 1:** Characteristics of articles, display items and *P* values by Journal-Year.

	Nature 2017	Nature 1997	Science 2017	Science 1997	PNAS 2017	PNAS 1997
Articles	65	84	63	66	54	58
Included articles*	65	78	62	61	51	54
Articles with *P* values in display items (% of included)	25 (38%)	23 (29%)	21 (34%)	9 (15%)	24 (47%)	8 (15%)
Articles describing multiplicity corrections (% of those with *P* values)	17 (68%)	1 (4%)	10 (48%)	0 (0%)	9 (38%)	1 (12%)
Display items in included articles	204	268	240	233	284	275
Display items with some (countable) *P* values	77 (74)	36 (36)	61 (60)	18 (18)	90 (87)	13 (12)
Total *P* values (median per display item)	564 (5.5)	224 (4)	751 (9)	151 (5)	797 (7)	55 (4)
Total significant *P* values (median per display item)	471 (5)	203 (4)	562 (6.5)	146 (5)	715 (6)	44 (3.5)

*excluding those with no display items, no data and reviews

### Total and statistically significant *P* values (Table 1, Fig 1, S1 and S2 Tables and S1 Fig)

Overall, the display items included 2542 countable *P* values, out of which 2141 were statistically significant. The use of *P* values escalated between 1997 and 2017, with 2.5 times more reported for Nature (224 versus 564), 5 times more for Science (151 versus 751) and 14.5 times more for PNAS (55 versus 797). A similar increase was mirrored by number of statistically significant *P* values: 2.3, 3.8 and respectively 16 times more for Nature (203 versus 471), Science (146 versus 562) and PNAS (44 versus 715). Additional descriptives are presented in S1 and S2 Tables.

**Fig 1:**
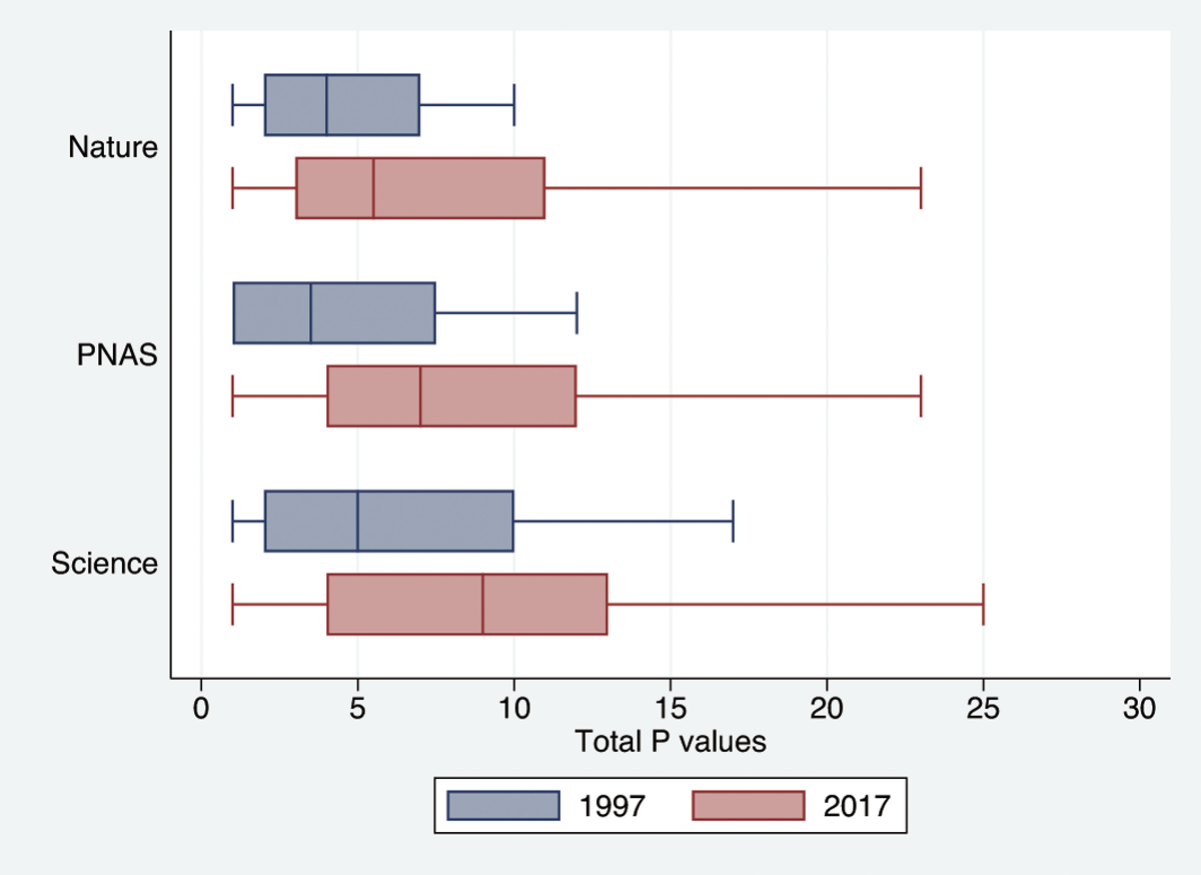
Box plots with standard errors for the total *P* values by Journal-Year cohorts. Eight outliers (> 30 *P* values), all from 2017, are not displayed. Median represented by a line on each bar.

### Proportion of statistically significant over total *P* values (Figs 2 and S2, S3, S4, S5, S6 and S7)

The pooled proportion of statistically significant *P* values across all Journal-Year units was 94.2 (95% CI 91.7% to 96.4%). Proportions for 2017 for Nature, Science and respectively PNAS were 92.5% (95% CI 86.4% to 95.6%), 87.5% (95% CI 79.8% to 93.9%) and 96.7% (95% CI 93.4% to 99.1%). Proportions for 1997 had smaller weights in the meta-analysis and were 98.5% (95% CI 93.7% to 100%), 100 (95% CI 99.9% to 100%), 90.1% (95% CI 75.1% to 99.4%) for Nature, Science and PNAS respectively. Differences among Journal-Year units were statistically significant (test of heterogeneity between subgroups: z = 25.99, p <.001).

**Fig 2:**
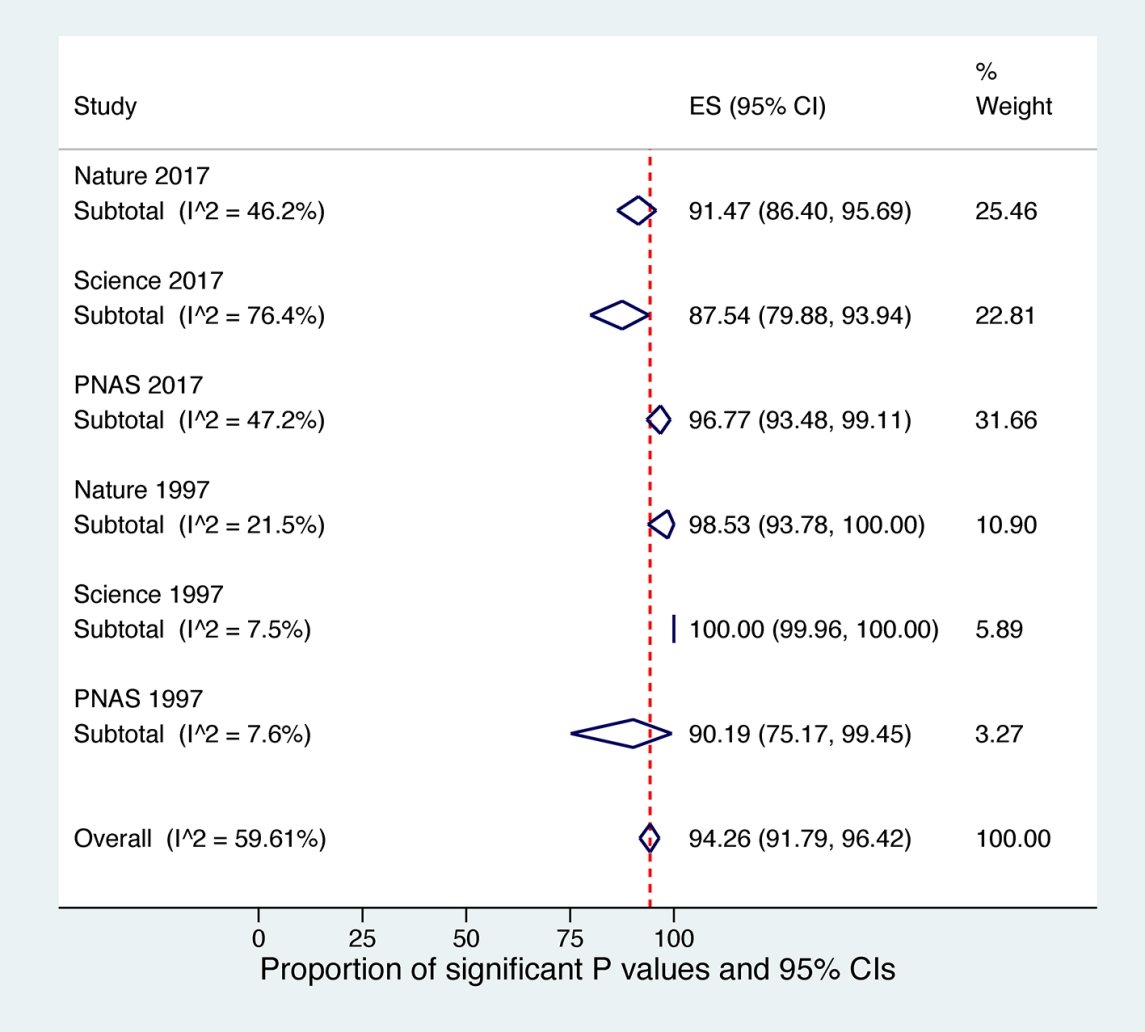
Proportion of significant P values and 95% confidence intervals All display items with countable P values by Journal-Year cohorts.

### Use of multiplicity corrections

Multiplicity corrections were described in 38 articles out of the 110 including display items with *P* values (34%, 95% CI 26% to 44%). In 2017, 36 out of 70 such articles (51%, 95% CI 39% to 63%) listed multiplicity adjustments: 17/25 (68%, 95% CI 46% to 85%), 10/21 (48%, 95% CI 26% to 70%) and 9/24 (38%, 95% CI 19% to 59%) for Nature, Science and, respectively, PNAS. Conversely, in 1997, only 2 articles (1 in Nature, 1 in PNAS) had described methods to correct for multiplicity.

### Bayesian methods

Ten articles out of the 390 that were eligible (2.5%, 95% CI 1% to 4.6%) used Bayesian statistical methods, and out of them, three (.7%, 95% CI .1% to 2%) did not include any *P* values. All of these articles were published in 2017 (4 in Nature, 3 in Science, and 3 in PNAS).

### Subgroup analyses

We first compared subgroups collapsing display items across journals. Pooled proportions were 92.6% (95% CI 89.6% to 95.3%) for 2017 and respectively 99.3% (95% CI 96.4% to 100%) for 1997. The difference between the two years did not survive Bonferroni multiplicity correction (test of heterogeneity between subgroups: z= 4.71, p= .03). The presence of multiplicity corrections was associated with clearly smaller proportion of statistically significant *P* values (z= 16.18, p<.001): proportions were 87.7%, 95% CI 82% to 92.6%, for articles mentioning corrections, and respectively 97.8%, 95% CI 96% to 99.2%, for articles not mentioning corrections. Sensitivity analyses restricted to articles published in 2017 indicated similar results: proportions 88.3, 95% CI 82.6% to 93.2%, for articles mentioning versus 96.1%, 95% CI 93.4% to 98.2%, for articles not mentioning corrections (z=10.27, p=0.001). Analysis by journal indicated no significant differences (z= 3.04, p=0.22).

In analysis stratified by year, the proportion of statistically significant to total *P* values in display items was not substantially different among the 3 journals in 2017 (z= 7.69, p= .021) and respectively 1997 (z= 11.90, p= .003), with the observed differences surviving the Bonferroni correction only for 1997.

Differences among the three journals were not substantial within the articles describing proper multiplicity corrections (z= 1.92, p= .38), and separately within the articles not describing these (z= 6.02, p=0.049).

### Robustness analysis (S1 Text, S8 Fig)

Some display items used atypical notations for statistical significance (e.g., bars with letter combinations), rendering them difficult to count and leaving some ambiguity. Fifteen display items, stemming from 5 articles, all in 2017, were in this situation (12 items from 4 articles in for Science and 3 items for 1 article for PNAS). For these 15 display items we applied rule (a) in 6 cases, rule (b) in 5 cases, and rule (c) in 5 cases. For 2 display items that were composed of various different panels with different indications, we used rule (a) for some panels and rule (c) for others. The available data in S1 Data indicate the exact articles and display items where each rule was applied. After excluding display items with atypical notations, results remained the same, with the exception of a slightly higher proportion of statistically significant *P* values for Science 2017 (90.8%, 95% CI 83.1% to 96.8%).

## Discussion

Our cross-sectional evaluation of *P* values reported in display items of three top science journals revealed a surge in their use over the last 20 years. Between 1997 and 2017, the number of display items containing *P* values increased between 2 and 7 times. It is unlikely the expansion was due to articles including more display items in general, as this figure remained largely unchanged. The use of *P* values across figures and tables proliferated even more prominently, with relative increases ranging from 2.5 to 14.5 times in the 3 assessed journals. The overwhelming majority of reported *P* values (94%) were statistically significant, in both years considered. Our findings dovetail with previous reports showing an increase in the reporting of *P* values over time and an almost ubiquitous occurrence of statistically significant results with very slow decrease over time [14]. Interestingly, this trend seems to be reversed in abstracts in epidemiology journals [44], where null hypothesis significance testing is becoming less popular. However, it is very probable that epidemiology (or some subdisciplines thereof) is a particular case, where some specific journals were ahead of the curve, maybe owing to the influence of methodologists who endorsed a more nuanced discussion of methods for statistical inference.

Reasons for the increase in total *P* values could be manifold, such as the proliferation of statistical analyses across papers, with *P* values being held as the norm for reporting these analyses [14], more powerful statistical software allowing for a myriad of analyses to be conducted and customization of figures so that more results can be displayed per figure. Reasons for the increase in statistically significant *P* values might reflect a genuine increment due to more variables being measured and more analyses being performed (hence augmenting the chance of obtaining lower *P* values) [14]. Non-null associations are likely to be abundant and more powerful methods or larger sample sizes might spuriously detect many of them as significant. However, it is likely that high prevalence of reported significant *P* values is a by-product of the highly competitive, “publish or perish”, academic culture, heavily incentivizing the delivery of “positive” (statistically significant) results, combined with suppression of “negative” (non-statistically significant) ones [51], through selective reporting [38, 52].

Corrections for multiplicity in articles containing display items with *P* values were almost non-existent in 1997, with only two articles that reported them. Methods to adjust for multiplicity were used far more in 2017, described in 37% to 68% of articles relying on *P* values in their display items. Undeniably, researchers can now count on unparalleled computational power, which also exposes them to the possibility to running a myriad of tests, making multiplicity corrections more important, particularly in fields such as genetics, neuroimaging and omics that are frequently published in the journals that we screened. Nonetheless, about half of the papers we surveyed and that reported any *P* values in display items did not describe any multiplicity corrections beyond those that are an intrinsic part of a classic statistical procedure, i.e. post-hoc tests for ANOVA, which we did not mark as necessarily satisfying adjustments since it is still possible to run myriads of ANOVAs in the same analysis.

Though there is vast scholarship on the scope, benefits and caveats of various methods to correct for multiple testing [21–28], the interrelated literature on the degree to which these methods are employed is more restricted. One survey [53] of conference abstracts in vision and ophthalmology found that out of the abstracts presenting *P* values, only 1.2% used some form of multiplicity correction. Moreover, a simulation study on the abstracts with no correction pointed to a false positive outcome in 30% of cases with more than 5 *P* values and almost 50% in the abstracts with more than 10 *P* values. The results of another meta-epidemiological assessment [54] showed that over 50% of multi-arm trials do not report using a correction for multiple testing. Finally, in an evaluation of two major orthopedic journals [55], authors found that corrections for multiple comparisons were present in 15% and respectively 6% of the articles that reported more than 5 *P* values from comparing two or more groups on a set of variables. The authors also calculated the Family Wise Error Rate (assuming independence between associations) and found it to be at 54% instead of the traditionally assumed 5%. Though our findings seem to provide a somewhat more optimistic assessment for papers published in 2017 in three top journals, we might have overestimated the adequacy of the multiplicity corrections used in relation to the study design and data. As we did not have the expertise and tools for an in-depth examination of all these extremely heterogeneous papers that cover the whole breadth of science, we coded for their presence as favorably as possible and considered any mention in the methods or display items (including supplementary) to be sufficient. Our experience in perusing these publications convinced us that it is indispensable for these papers to be peer-reviewed not only by field experts, but also by statistical experts who work specifically in the same field, before their publication in these extremely prestigious venues.

In the absence of multiplicity corrections, almost all reported *P* values were statistically significant (98%). Conversely, if multiplicity corrections were described, 88% of reported *P* values were statistically significant. This is still a very high proportion, but it is at least less suggestive of reported results being almost universally statistically significant. Differences in the proportions of statistically significant *P* values between corrected and uncorrected articles were statistically significant in both the main analysis and sensitivity analysis. Moreover, differences among journals within the corrected and respectively uncorrected strata were small and generally not beyond chance.

Use of Bayesian methods was scant and for the most part they were used in articles that also included *P* values. Under 1% of the articles screened relied exclusively on Bayesian statistics. However, as all these articles were published in 2017, this finding might reflect a genuine (even if small in absolute magnitude) increment in the uptake of these methods. A case for the use of Bayesian methods has been frequently made in the methodological literature [6, 29], potential benefits for their application to various strains of basic [31–33] and applied [34–36] research have been demonstrated, and a growing number of methodological resources [37] and software packages (e.g., JASP, Stan, WinBUGS) for their implementation exist. Yet the uptake of these methods in research published in top journals remains extremely low.

There are several limitations to our study. As a cross-sectional design, differences between 1997 and 2017 are purely observational, subject to many potential confounding variables. Research published 20 years apart may be very different, including in its genuine needs to correct for multiplicity. However, our goal was not to look at causes or predictors in the evolution of *P* values. Rather, we were interested in two descriptive snapshots over a 20-year period, as potential indication of whether scientists and top journals have undergone a paradigm shift in relation to statistical testing. Another possible limitation of our analysis is that we have used two time points only (1997 and 2017) and thus we cannot identify the exact temporal trends for the changes that happened over these twenty years in the use of P-values. Our assessment required very intensive manual extraction of data, as opposed to previous assessments of *P* values in the text that capitalized on automated text mining and could thus examine continuous time trends. However, it is likely that the observed changes in patterns, e.g. use of multiplicity adjustments, are likely to have happened in a continuous fashion throughout the 20-year period rather than reflect a sudden, acute change in use of statistical inference tools and methods.

Journal-Year units were searched in a sequential way that was pre-specified by us rather than chosen at random. It was also not feasible to blind the journal and year during assessment. While this may create risk of bias, the outcome was highly objective (based on count of P values) and data extraction is unlikely to be influenced by knowledge of journal and year and by knowledge of the data in previously assessed Journal-Year units. A minority of ambivalent, complex cases where counting was unsure were discussed between the two authors and subjected to robustness analyses. In some types of graphic elements, such as scatter plots, Manhattan plots, volcano plots, brain activation maps, heat maps and others, the number of *P* values was impossible to count manually. In these cases, we used estimations of total *P* values based on other panels of the figure or, if nothing was countable, excluded it completely. This undoubtedly underestimated the number of *P* values in 2017, which is probably considerably larger. The proportions of statistically significant *P* values are clearly much lower when all the data are presented in such a figure. Nevertheless, these comprehensive full-data figures represented a very small minority even in 2017.

We extracted information about the use of corrections from the Methods section, both in the main paper and supplementary material, as well as from the display items screened or those present in the supplementary material. However, unless the authors specifically mentioned correction methods and identified them as such at least by naming the procedure, we could not ascertain whether such a method was used. Hence, it is possible that we wrongly coded as uncorrected some articles that had in fact performed corrections and only specified the name of the programs used to analyze data, or used vague, all-purpose statements like “analyses were performed as previously described” or “according to standard protocols”. This confusion would have been fully dissipated in the presence of complete and accurate reporting of statistical tests performed, as recommended by several reporting guidelines (e.g., ARRIVE for animal studies [56]). Moreover, statistical reporting seemed particularly elliptic in the type of basic [57], frontier research that journals like Nature, Science and PNAS predominantly publish. Conversely, we focused only on capturing whether any effort was made for multiplicity correction, without judging whether this method was appropriate and properly applied on the data. Such a decision would have required in-depth expertise in all the multifarious fields covered by the assessed papers and full access to the protocols and data. Therefore, the proportion of papers that not only use multiplicity corrections, but use them fully appropriately, is likely to be smaller than what we recorded. Moreover, since the multiplicity correction method was seldom described in the display item, we could not match it to each of the *P* values extracted. For example, the method of correction might have been used for one section of the analyses, which might not have been germane to all the display items with *P* values in the article. Consequently, not all *P* values stemming from what we labelled as corrected figures are necessarily corrected, and thus we probably underestimated the number of uncorrected *P* values. In all, multiplicity corrections seem to continue to be under-utilized even in 2017.

Display items in the three top multi-disciplinary science journals rely heavily on *P* values, the use of which has grown fast over the last 20 years. Overwhelmingly and almost universally, *P* values in display items point to statistically significant results. Importantly, significance testing and *P* values could only inform the existence of associations, but as spurious associations are almost ubiquitous, the real scientific challenge is to distinguish those that are most relevant for causal inference. The use of multiplicity corrections is one factor that makes a small dent by attenuating the ubiquitous prevalence of statistically significant *P* values. Though a profound paradigm shift in relation to statistical testing is often forecast and advocated, such a shift does not seem to have hitherto permeated publications in top science journals. More widespread use of multiplicity corrections, particularly in fields where a myriad of tests is customarily run, and use of different inferential methods besides significance testing still need to be promoted.

## Supporting information

S1 Text Robustness analysis. (DOCX)

S1 Table Descriptive statistics for the total number of countable *P* values across display items for each Journal-Year unit. (DOCX)

S2 Table Descriptive statistics for the total number of significant *P* values across display items for each Journal-Year unit. (DOCX)

S1 Fig Box plots with standard errors for the total significant *P* values by Journal-Year cohorts. Four outliers (> 30 P values), all from 2017, are not displayed. Median represented by a line on each bar.
(TIF)

S2 Fig Proportion of significant *P* values and 95% confidence intervals across all display items with countable *P* values in Nature 2017. (TIF)

S3 Fig Proportion of significant *P* values and 95% confidence intervals across all display items with countable *P* values in Nature 1997. (TIF)

S4 Fig Proportion of significant *P* values and 95% confidence intervals across all display items with countable *P* values in Science 2017. (TIF)

S5 Fig Proportion of significant *P* values and 95% confidence intervals across all display items with countable *P* values in Science 1997. (TIF)

S6 Fig Proportion of significant *P* values and 95% confidence intervals across all display items with countable *P* values in PNAS 2017. (TIF)

S7 Fig Proportion of significant *P* values and 95% confidence intervals across all display items with countable *P* values in PNAS 1997. (TIF)

S8 Fig Robustness analysis. Proportion of significant *P* values and 95% confidence intervals by Journal-Year cohorts excluding 15 display items (all in 2017) with uncertainty about counting.
(TIF)

S1 Data Extracted data for all screened articles. (XLSX)
