# Supplementary material for: P values in display items are ubiquitous and almost invariably significant: A survey of top science journals
Source: PLoS One. 2018 May 15;13(5):e0197440. doi: 10.1371/journal.pone.0197440 (PMC5953482; doi:10.1371/journal.pone.0197440)
Supplement: S1 Text — (DOCX) [file pone.0197440.s001.docx]

**Robustness analysis**

For 15 display items, stemming from 5 articles (12 items in 4 articles for Science 2017 and 3 items in 1 article for PNAS 2017), using atypical methods of flagging statistical significance (usually combinations of letters on bar graphs), we were unsure whether our method of counting *P* values correctly reflected the comparisons underpinning the graph. Therefore, we checked the robustness of our findings by running sensitivity analyses excluding these problematic items.

**Proportion of significant to total P values (S8 Fig)**

The pooled proportion across all Journal Year units was 95.3% (95% CI 93% to 97%). Prevalence rates for 2017 for Nature, Science and respectively PNAS were 91.4% (95% CI 86.4% to 95.6%), 90.8% (95% CI 83% to 96.8%) and 97.2 (95% CI 93.9% to 99.4%). Prevalence rates for 1997 remained unchanged given there were no display items with uncertainty about the counting of P values in this year. Differences among Journal Year units were significant (test of heterogeneity between subgroups: z= 23.03, p<.001)

**Subgroup analyses**

Collapsing display items across journals, the presence of adequate correction methods described in the articles was associated with significant differences between subgroups (test of heterogeneity between subgroups: z= 17.17, p<.001). Prevalence rates were 88.8%, 95% CI 83.3% to 93.6%, for articles mentioning corrections, versus 98.5%, 95% CI 96.9% to 99.6%, for articles not mentioning corrections. Analyses restricted to articles published in 2017, which included the bulk of multiplicity corrections, revealed similar proportions for corrected (89.5%, 95% CI 84.1% to 94.2%) versus uncorrected articles (97.1%, 95% CI 94.6% to 99%) and differences between the two subgroups remained significant (z=11.45, p=0.001), surviving the Bonferroni corrected threshold.

Prevalence rates did not differ between articles published in 2017 (93.9%, 95% CI 91.1% to 96.3%) and 1997 (99.3%, 95% CI 96.4% to 100% in 1997), z= 3.17, p= .075 or among the three journals, z= 2.30, p= .32. Differences among journals in the proportion of significant to total *P* values bordered statistical significance and did not survive the Bonferroni correction for 2017 (z= 6.06, p= .048) ad. For 1997, analyses were unaffected by uncertainly about counting.

Analyses focusing on the use of multiplicity corrections, restricted to 2017, showed non-significant differences among the three journals, both within articles describing corrections (z= 1.15, p= .56) as well as for those not mentioning them (z= 3.67, p= .16)
