# Supplementary material for: P values in display items are ubiquitous and almost invariably significant: A survey of top science journals
Source: PLoS One. 2018 May 15;13(5):e0197440. doi: 10.1371/journal.pone.0197440 (PMC5953482; doi:10.1371/journal.pone.0197440)
Supplement: S1 Table — (DOCX) [file pone.0197440.s002.docx]

**S1 Table. Descriptive statistics for the total number of countable *P* values across display items for each Journal-Year unit**

| Total *P* values | N | Sum | Min | Max | Median (per display item) | IQR |
| --- | --- | --- | --- | --- | --- | --- |
| Nature 2017 | 74 | 564 | 1 | 32 | 5.5 | 8 |
| Nature 1997 | 36 | 224 | 1 | 30 | 4 | 5 |
| Science 2017 | 60 | 751 | 1 | 133 | 9 | 9 |
| Science 1997 | 18 | 151 | 1 | 27 | 5 | 8 |
| PNAS 2017 | 87 | 797 | 1 | 54 | 7 | 8 |
| PNAS 1997 | 12 | 55 | 1 | 12 | 3.5 | 6.5 |
