# Supplementary material for: P values in display items are ubiquitous and almost invariably significant: A survey of top science journals
Source: PLoS One. 2018 May 15;13(5):e0197440. doi: 10.1371/journal.pone.0197440 (PMC5953482; doi:10.1371/journal.pone.0197440)
Supplement: S2 Table — (DOCX) [file pone.0197440.s003.docx]

**S2 Table. Descriptive statistics for the total number of significant *P* values across display items for each Journal-Year unit**

| Significant *P* values | N | Sum | Min | Max | Median (per display item) | IQR |
| --- | --- | --- | --- | --- | --- | --- |
| Nature 2017 | 74 | 471 | 1 | 25 | 5 | 6 |
| Nature 1997 | 36 | 203 | 1 | 26 | 4 | 5 |
| Science 2017 | 60 | 562 | 1 | 59 | 6.5 | 9 |
| Science 1997 | 18 | 146 | 1 | 27 | 5 | 9 |
| PNAS 2017 | 87 | 715 | 1 | 54 | 6 | 9 |
| PNAS 1997 | 12 | 44 | 1 | 8 | 3.5 | 4 |
