# Supplementary figures and images for: P values in display items are ubiquitous and almost invariably significant: A survey of top science journals
Source: PLoS One. 2018 May 15;13(5):e0197440. doi: 10.1371/journal.pone.0197440 (PMC5953482; doi:10.1371/journal.pone.0197440)

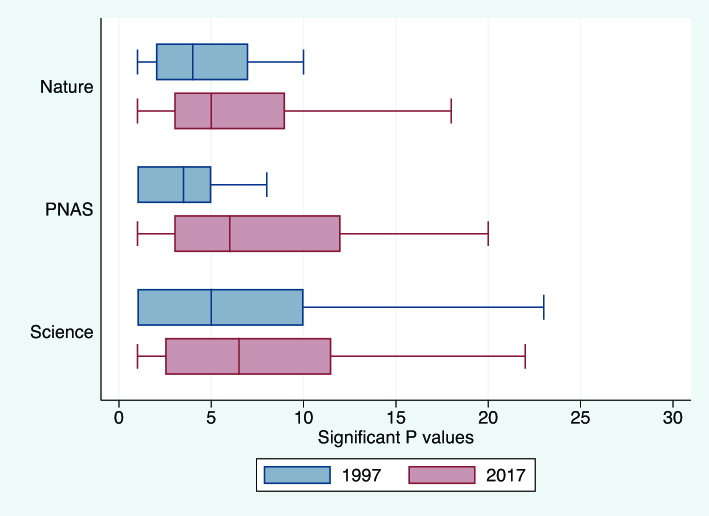

Supplement: S1 Fig — Four outliers (> 30 P values), all from 2017, are not displayed. Median represented by a line on each bar. (TIF) [file pone.0197440.s004.tif]

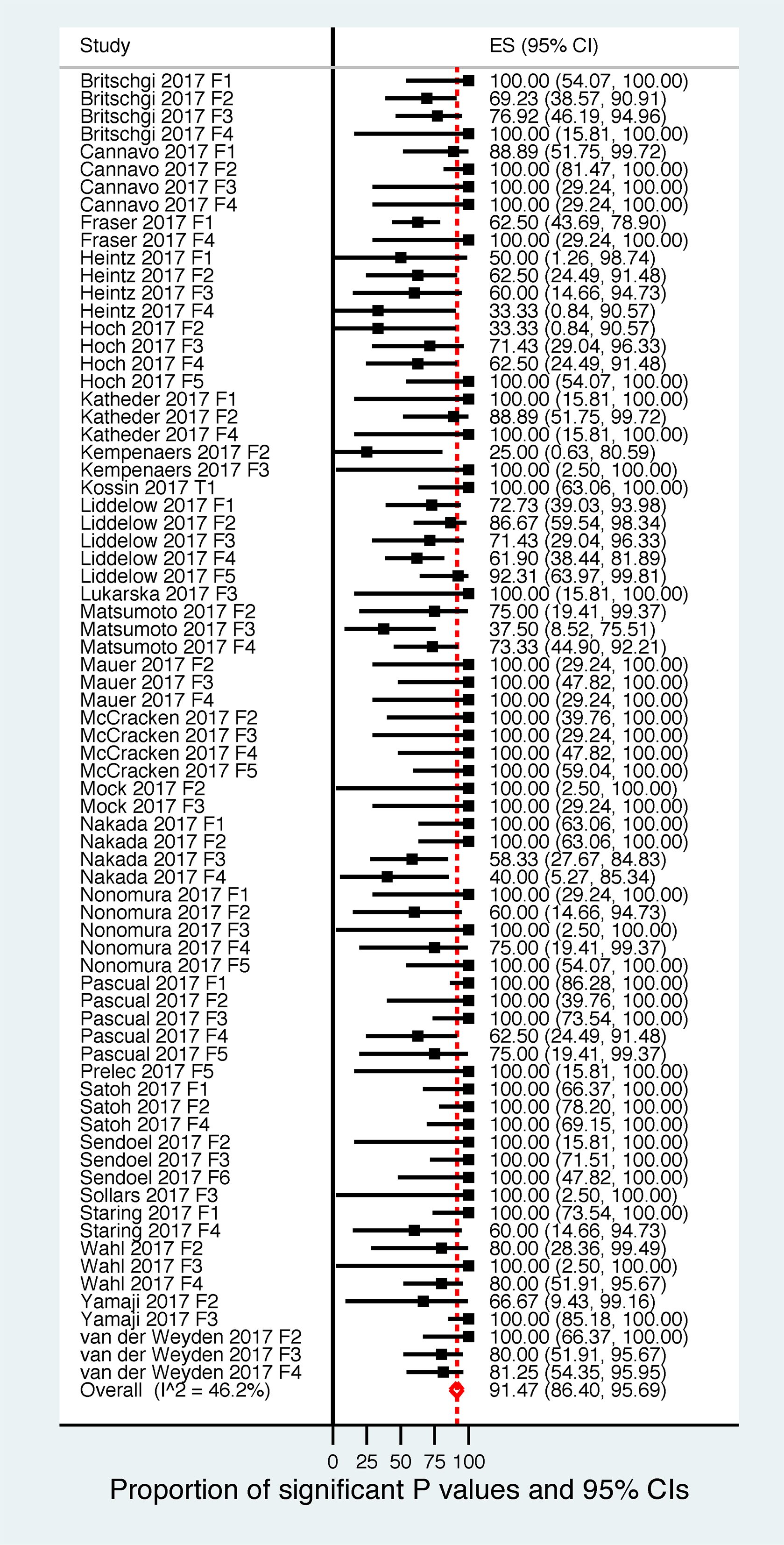

Supplement: S2 Fig — (TIF) [file pone.0197440.s005.tif]

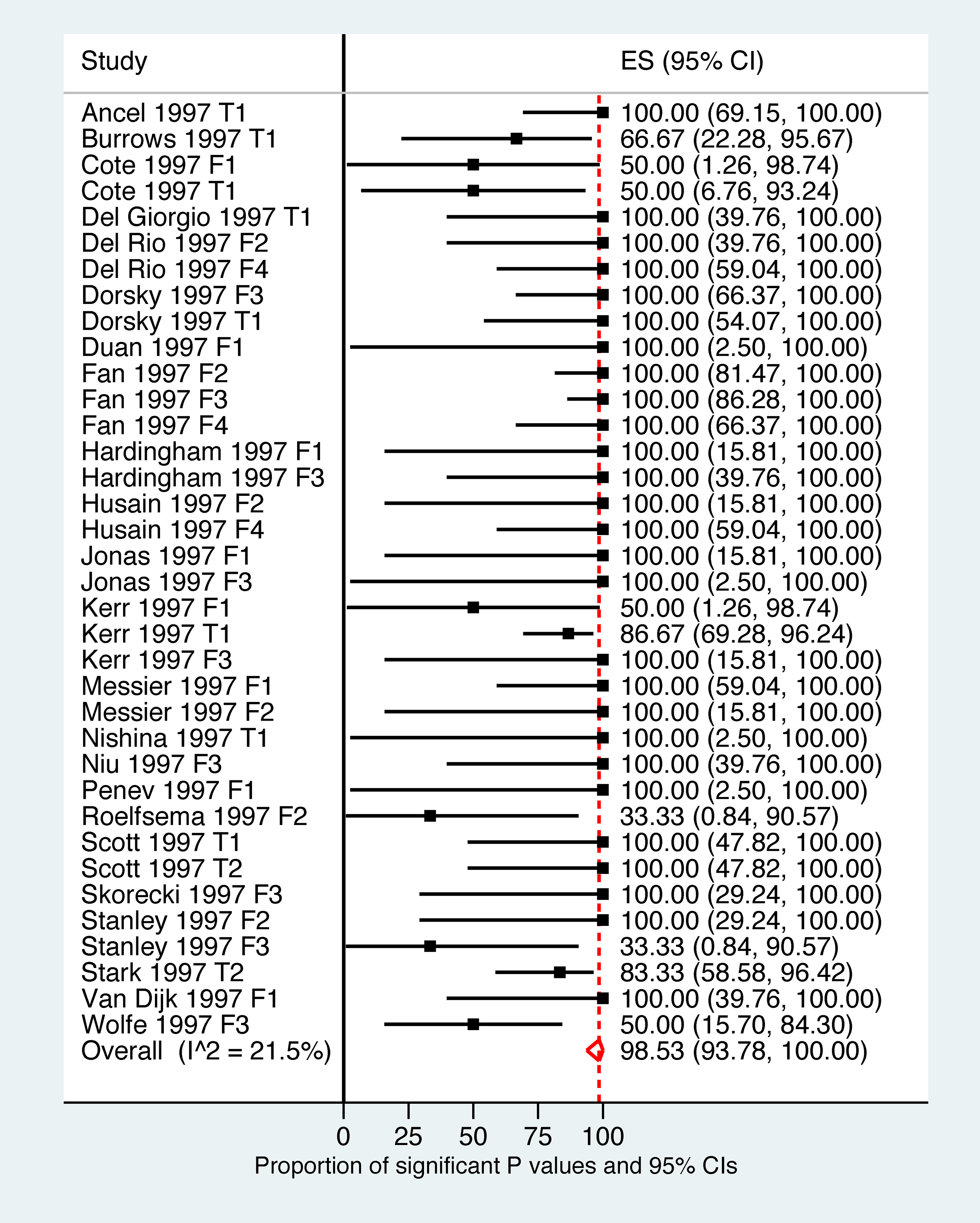

Supplement: S3 Fig — (TIF) [file pone.0197440.s006.tif]

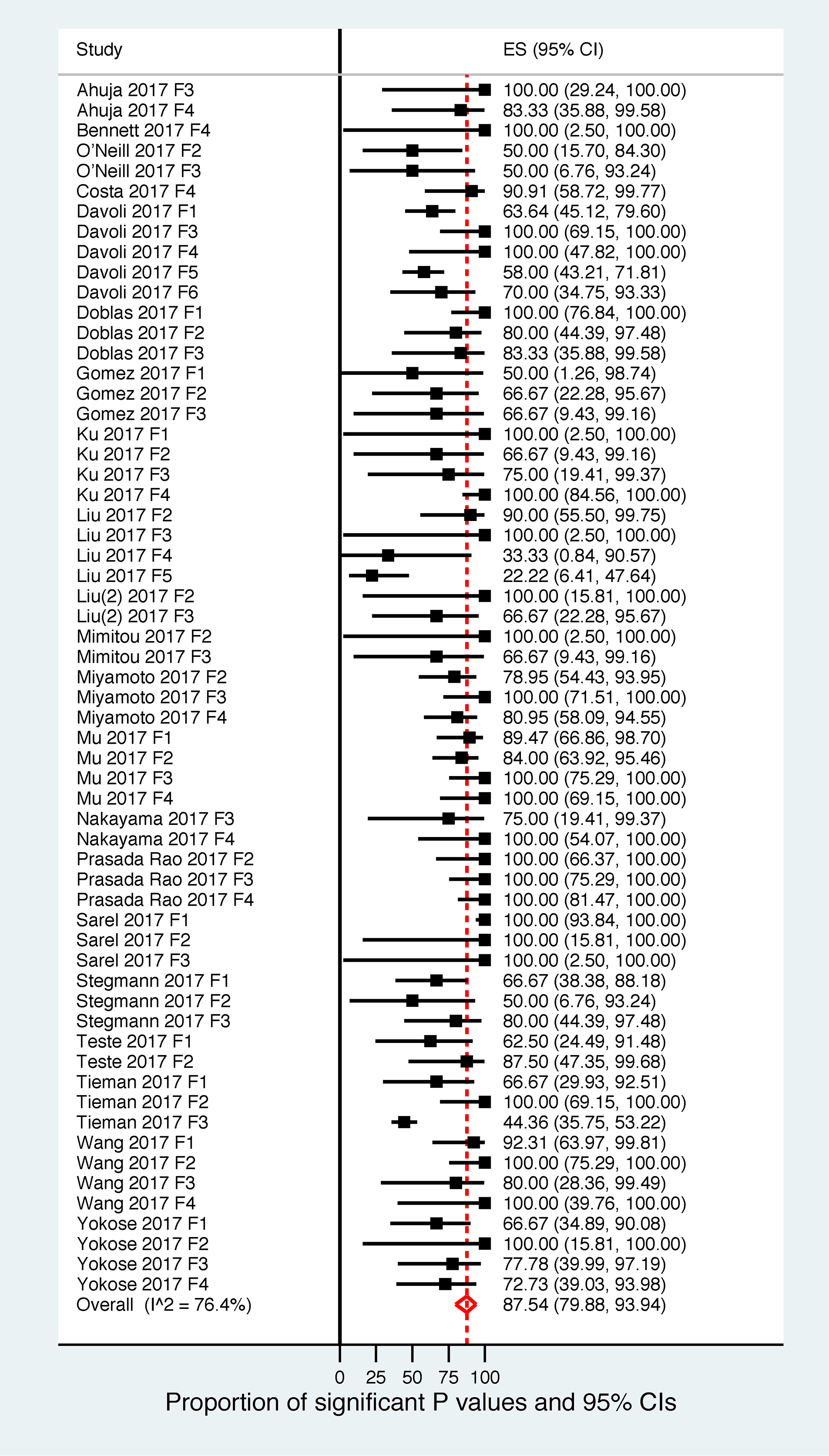

Supplement: S4 Fig — (TIF) [file pone.0197440.s007.tif]

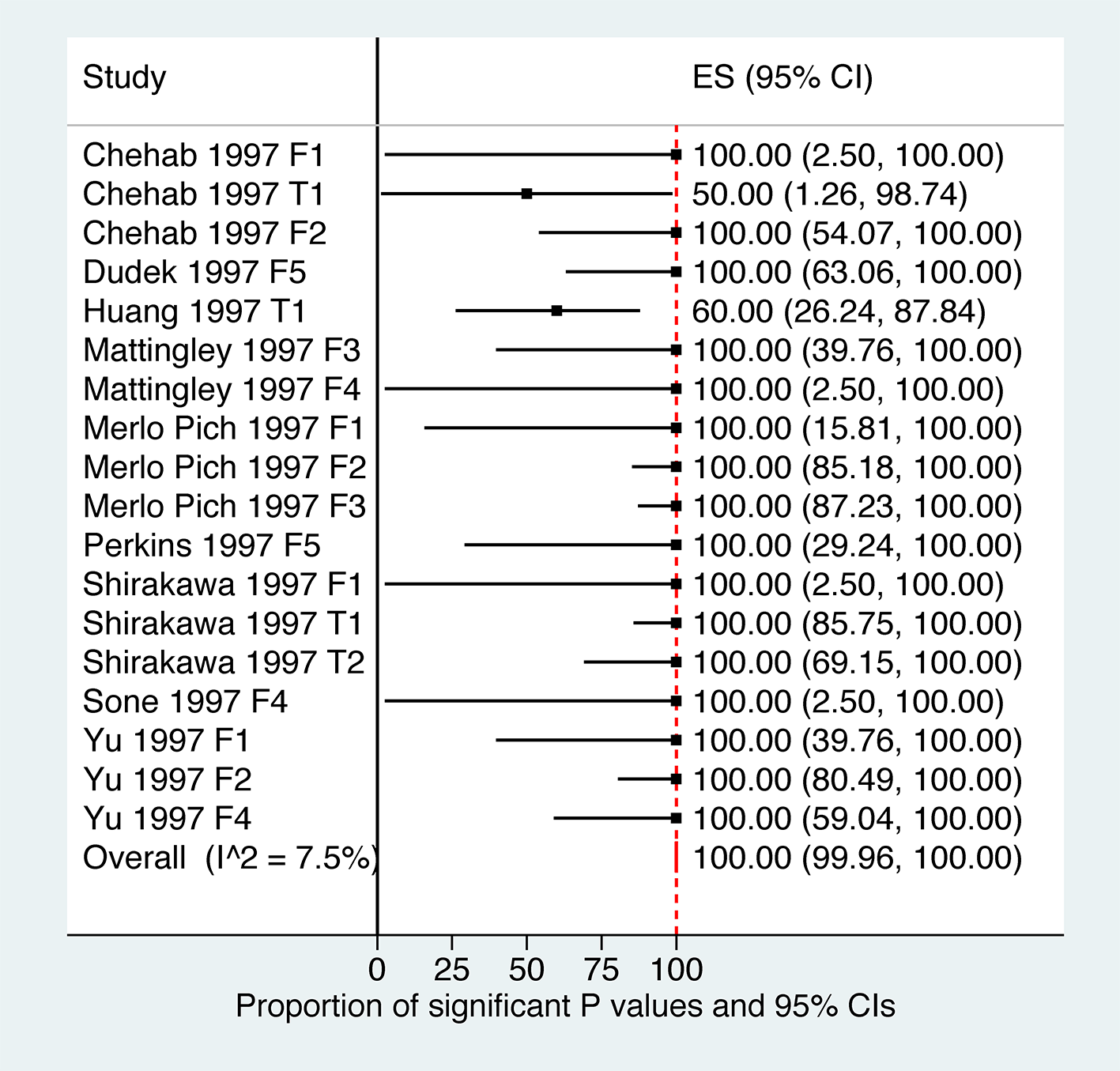

Supplement: S5 Fig — (TIF) [file pone.0197440.s008.tif]

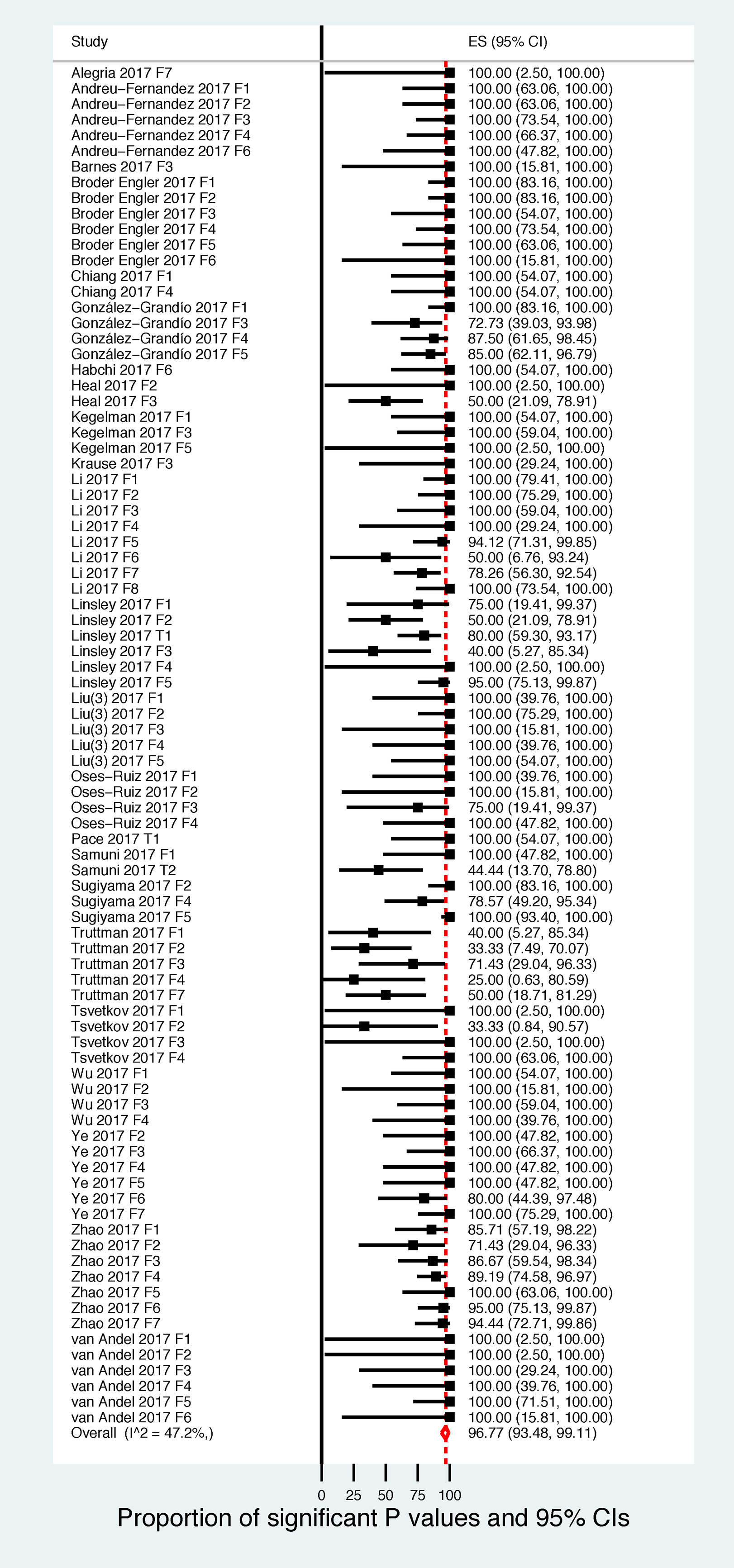

Supplement: S6 Fig — (TIF) [file pone.0197440.s009.tif]

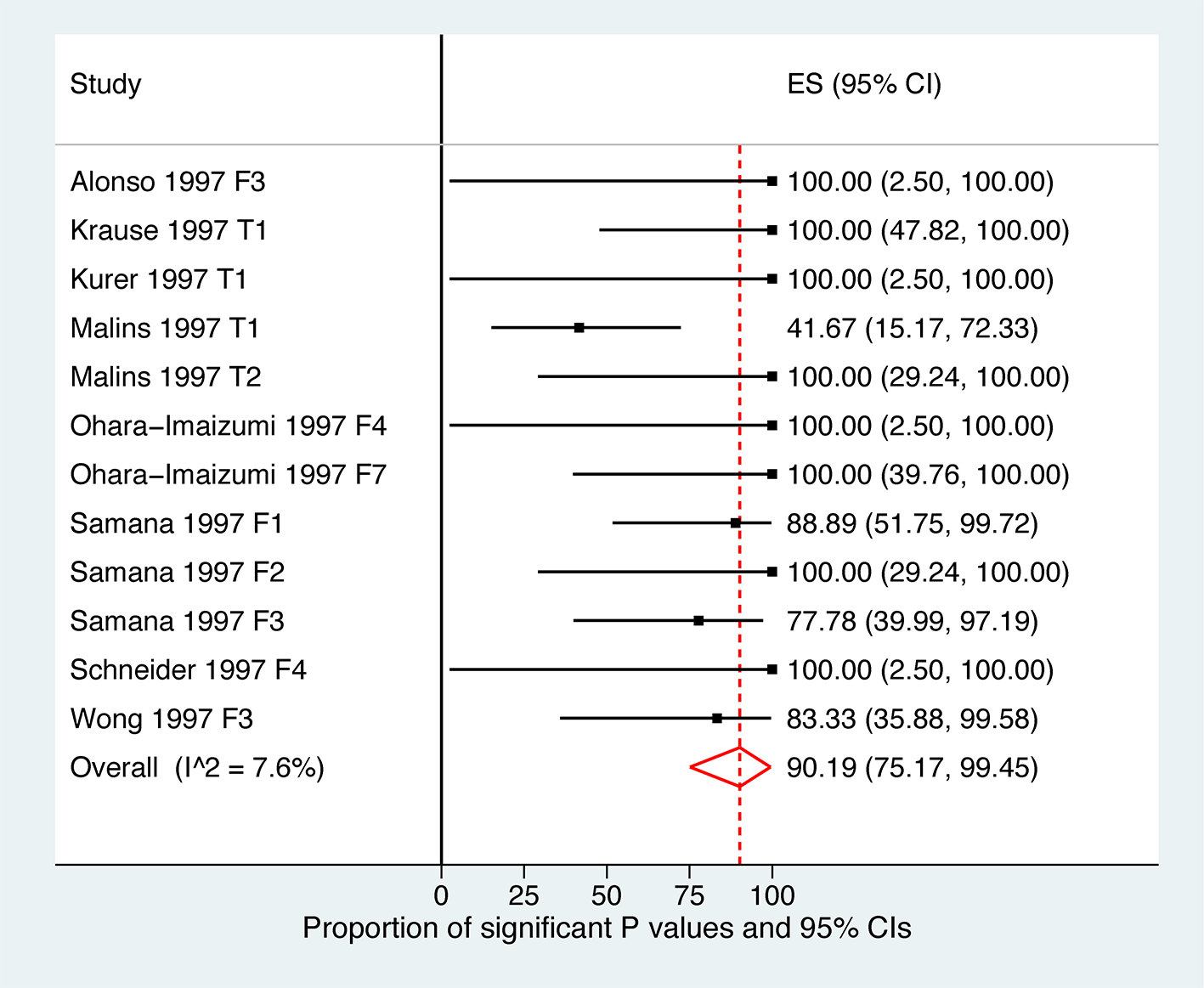

Supplement: S7 Fig — (TIF) [file pone.0197440.s010.tif]

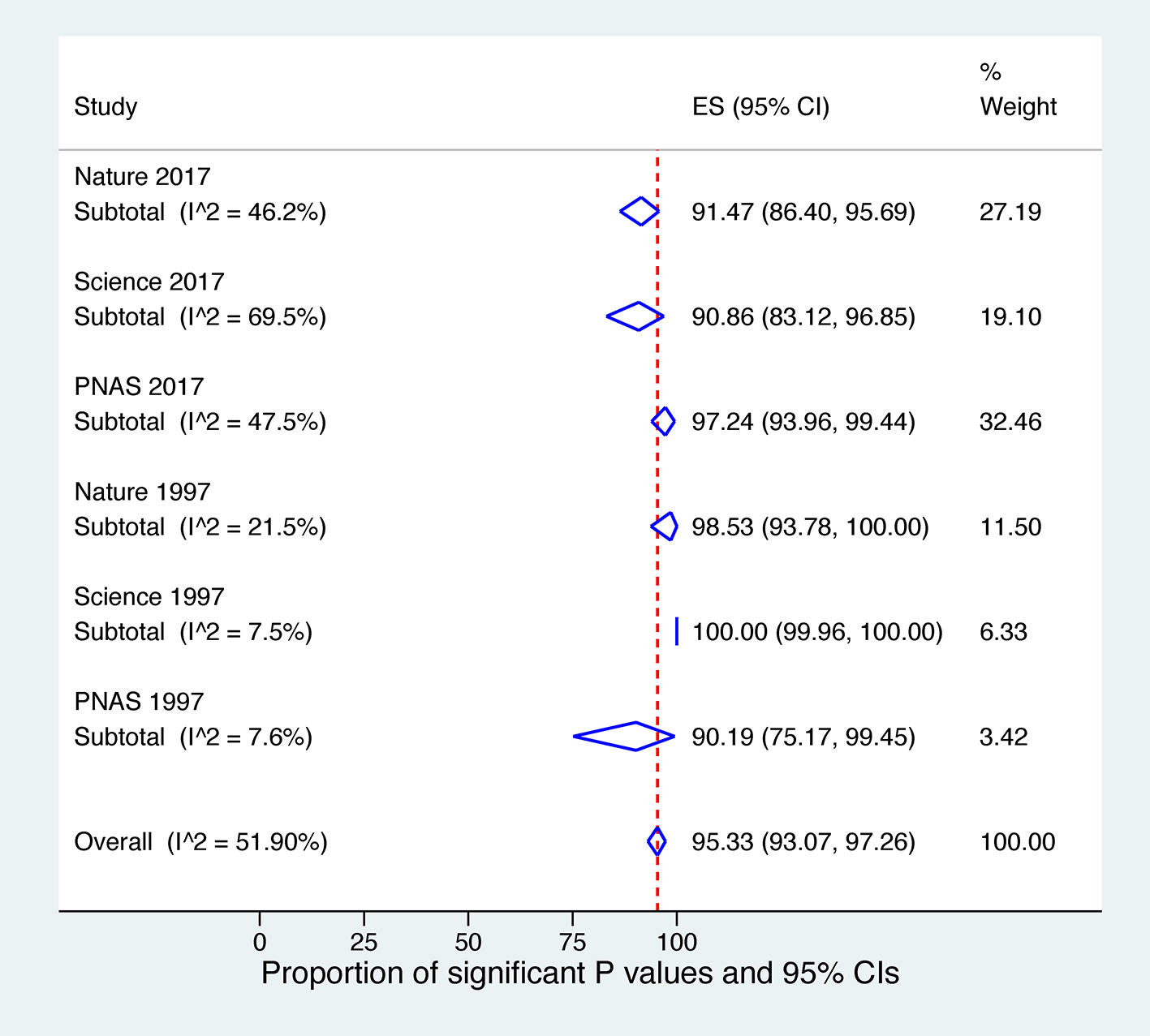

Supplement: S8 Fig — Proportion of significant P values and 95% confidence intervals by Journal-Year cohorts excluding 15 display items (all in 2017) with uncertainty about counting. (TIF) [file pone.0197440.s011.tif]
